# Experimental Microwave Scattering in Polygonal Billiards

**DOI:** 10.1038/s41598-019-40048-0

**Published:** 2019-03-06

**Authors:** R. B. do Carmo, F. M. de Aguiar

**Affiliations:** Universidade Federal de Pernambuco, Departamento de Física, Recife, PE 50670-901 Brazil

## Abstract

Fluctuations in the one-port scattering and normalized impedance matrices in three polygonal and one chaotic time-reversal invariant microwave billiards are experimentally investigated, in several levels of coupling and absorption, at room temperature and at 77 K. The observed distributions of reflection coefficient, phase of the scattering matrix, resistance and reactance exhibit no fingerprint of a given geometry. At low frequencies, the results are consistent with earlier theoretical models by López, Mello and Seligman and by Zheng, Antonsen and Ott, who independently predicted that the scattering fluctuations might be the same for the Wigner and Poisson level spacing distributions in the lossless cavity. The uniqueness of the observed scattering statistics at higher absorption levels is discussed with respect to inherent limitations posed by the experimental technique.

## Introduction

Scattering plays a prominent part in the development of physics and engineering. In the past few decades, universal properties of chaotic scattering have been vigorously investigated in nuclei^1^, ballistic electronic cavities^2^ and microwave billiards^3,4^. In general, the scattering process has a stochastic nature due to the complexity of the interaction between the target and the projectiles, allowing for statistical descriptions of the phenomenon. Microwave billiards enjoy an interesting electromagnetic analogy with two-dimensional quantum wells and offer advantages for experimental tests of predictions based on random matrix theory (RMT). Experiments in these flat resonators were introduced in the early 1990’s as a tool in the study of quantization of classically chaotic systems^5^. In 1990, Doron, Smilansky and Frenkel^6^ reported the first experimental studies of microwave chaotic scattering. A number of investigations on the spectral properties of *closed* systems appeared in the literature in the following decade, but we had to wait a bit longer for new experimental data on *scattering* properties of *open* systems. For a time-reversal invariant target in the one-port case, two independent approaches have been reported in the past decade, one for the scattering matrix *S*, the other one for the impedance matrix *Z*. These matrices are related through $Z/{Z}_{0}=(1+S)/(1-S)$, where *Z*_0_ is the characteristic impedance of the transmission line that connects the microwave source with the target. *S* is usually expressed as the complex number $S=\sqrt{R}{e}^{i\theta }$, where *R* is the reflection coefficient. In 2003, measurements of fluctuations in *R* were reported in half Sinai and disordered billiards by Méndez-Sánchez *et al*.^7^. Distributions of the phase *θ* in the same cavities appeared a couple of years later^8^. The measured distributions *P*(*R*) and *P*(*θ*) were successfully interpreted with a model for which the joint distribution is given by^8^

1 $$P(R,\theta ;\gamma ,t)=\frac{1}{2\pi }{(\frac{1-{\langle S\rangle }^{2}}{|1-S\langle S\rangle {|}^{2}})}^{2}P(R,\theta ;\gamma ,1),$$

where *γ* is the absorption parameter and $t=1\,-\,{\langle S\rangle }^{2}$ is the coupling parameter. Integration of $P(R,\theta ;\gamma ,t)$ over *θ* gives *P*(*R*) and through a similar projection over *R* one obtains the theoretical phase distribution *P*(*θ*). The ideal distribution $P(R,\theta ;\gamma ,1)$ (perfect coupling) has well defined limits for $\gamma \ll 1$ and $\gamma \gg 1$. Otherwise, heuristic interpolation formulas have been proposed^8,9^. Universal fluctuations in *Z* were independently studied in a quartered bow tie billiard^10,11^. The *Z* measurements also aimed at testing predictions of RMT^12^, assuming chaotic properties of the target. More precisely, the theory considers the normalized impedance $z=(Z-i{\rm{Im}}[{Z}_{rad}])/\mathrm{Re}[{Z}_{rad}]$, where *Z*_*rad*_ is the radiation impedance, *i*.*e*., the impedance seen at the antenna when the transmission line is coupled to a two-dimensional cavity with the same thickness but the side walls displaced to infinity. No transmitted wave returns to the antenna in this hypothetical structure, which can be experimentally realized by covering the side walls of the cavity with strips of a broadband microwave absorber. Let ${\rm{Re}}[z]={z}_{R}$ be the normalized resistance and ${\rm{Im}}[z]={z}_{I}$ the normalized reactance. Predictions of the random wave model^12^ for the *lossless* cavity include: (i) The impedance statistics are the same for both Poisson and Wigner distributions of the nearest neighbor spacing in the unfolded spectrum of squared eigenfrequencies, and are not affected by long range spectral correlations; (ii) ${z}_{R}=0$; (iii) *P*(*z*_*I*_) is Lorentzian. In the presence of losses, *P*(*z*_*R*_) exhibits an asymmetric peak which increases and shifts to the right as damping is increased, whereas *P*(*z*_*I*_) exhibits a remarkable transition from a Lorentzian (low loss) to a Gaussian (high loss) distribution, also consistent with previous calculations^9^. With normalization, the impedance approach avoids the dependence on coupling, so that the measured distributions could be successfully fitted simultaneously with a single damping parameter in Monte Carlo simulations^11,12^. It is interesting to notice that prediction (i) for the cavity with no loss bears a relation to an earlier result reported by López, Mello and Seligman (LMS) in the early 1980’s^13^, in the context of statistical nuclear physics. LMS considered an ensemble of matrices $S={e}^{i\theta }$ obeying the so-called “analiticity-ergodicity” requirement, for which the phase distribution is given by

2 $${P}_{{\rm{LMS}}}(\theta )=\frac{1}{2\pi }\frac{1\,-\,|\langle S\rangle {|}^{2}}{1+|\langle S\rangle {|}^{2}\,-\,2{\rm{Re}}\,(\langle S\rangle {e}^{i\theta })},$$

where $\langle S\rangle$ is the average of *S*, independently of the statistical law proposed for the energy spectrum. In particular, LMS demonstrated numerically that correlated and uncorrelated spectra lead to one-channel scattering processes uniquely described by ${P}_{{\rm{LMS}}}(\theta )$^13^. Let *s* be the nearest neighbor spacing. The correlated spectrum was described by the Wigner surmise

3 $${P}_{{\rm{W}}}(s)=\frac{\pi }{2}s\,\exp \,(-\frac{\pi }{4}{s}^{2}),$$

whereas the uncorrelated spectrum was characterized by the Poisson distribution

4 $${P}_{{\rm{P}}}(s)=\exp \,(\,-\,s).$$

**Figure 1:**
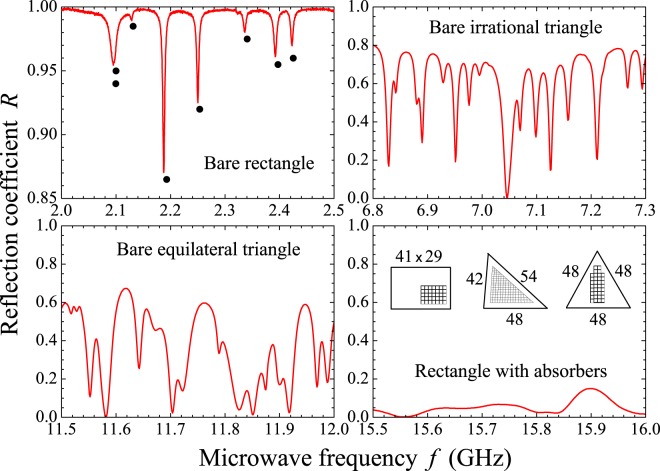
Solid red lines: Measured reflection coefficient *R* as a function of frequency *f*, in several regimes of coupling and absorption in the indicated billiards. A *bare* billiard means a billiard with no added absorber. Lower right inset: In-plane geometries of the three billiards used in the experiments. Numbers are dimensions in cm. The shaded areas indicate the regions swept by the antenna position for average purposes. The dots in the upper left panel indicate positions of the exactly calculated resonances (see also Figs 11, 12 and 17).

In fact, *P*_W_(*s*) and *P*_P_(*s*) became paradigmatic functions in the field of “quantum chaos”, the former associated with classically chaotic systems, the latter with integrable ones. In regard to *scattering*, nearly all experiments hitherto reported in the literature consider in-plane geometries with a *chaotic* classical limit. Here we report results of a thorough experimental study in the 2–18 GHz frequency interval in three *polygonal* microwave billiards with distinct semiclassical spectral fluctuations. Billiards in polygons are known to have zero Kolmogorov-Sinai entropy and, therefore, are not chaotic. However, there are numerical evidences for the mixing property in certain irrational triangles^14,15^ whose quantization displays spectral fluctuations that approach those of the Gaussian orthogonal ensemble (GOE) of random matrices. Thus, polygons offer the opportunity for scattering experiments in billiards with varying semiclassical spectral statistics, such as the rectangle, the irrational triangle and the equilateral triangle studied here. Geometry and dimensions are sketched in the inset of the right bottom panel in Fig. 1. The shaded areas correspond to the regions scanned by the antenna for average purposes. Places near the cavity border were not used due to poor coupling for all modes. In addition, when there is a symmetry in the billiard, care was taken not to choose equivalent antenna positions. The (42 cm, 48 cm, 54 cm) billiard is an experimental realization of an irrational triangle (all angles irrational with *π*) which is classically *mixing* and exhibits GOE quantum spectral fluctuations, according to numerical calculations^15,16^. We also report comparative results in the chaotic Sinai billiard. Each resonator investigated here has a depth *d* = 6.0 mm, so that all resonating TM modes are two-dimensional for a frequency $f < c/2d=25\,{\rm{GHz}}$. A single monopole antenna with height $h=4.1\,{\rm{mm}}$ and radius $r=0.47\,{\rm{mm}}$ is used (see Fig. 11). A primary characterization of the spectra investigated is given in Table 1, where we show the calculated values of the ground state frequency (*f*_1_), the lowest (*f*_*L*_) and highest (*f*_*H*_) eigenfrequencies in the 2–18 GHz experimental range, the corresponding free-space wavelength $({\lambda }_{L,H}=c/{f}_{L,H})$, the number of modes below 2 GHz (*N*_*L*_), the number of modes below 18 GHz (*N*_*H*_), and the number of modes Δ*N* in the 2–18 GHz frequency range.

**Table 1:** Calculated parameters of the billiards experimentally investigated in this work: *f*_1_ is the ground state frequency, *f*_*L*_ (*f*_*H*_) is the lowest (highest) eigenfrequency in the 2–18 GHz interval accessed in the experiments, *λ*_*L*_ (*λ*_*H*_) the corresponding free space wavelength, *N*_*L*_ (*N*_*H*_) is the number of resonant modes below 2 GHz (18 GHz), and Δ$N={N}_{H}-{N}_{L}$.

Billiard	*f*_1_ (GHz)	*f*_*L*_ (GHz)	*f*_*H*_ (GHz)	*λ*_*L*_ (cm)	*λ*_*H*_ (cm)	*N*_*L*_	*N*_*H*_	Δ*N*
Rectangle	0.6331	2.0996	17.9998	14.2785	1.6655	12	1306	1294
Equilateral triangle	0.4164	2.0819	17.9912	14.4000	1.6663	14	1127	1113
Irrational triangle	0.7411	2.0327	17.9972	14.7484	1.6658	9	1048	1039
Sinai	0.8342	2.0821	17.9992	14.3987	1.6656	10	1220	1210

## Results

**Figure 2:**
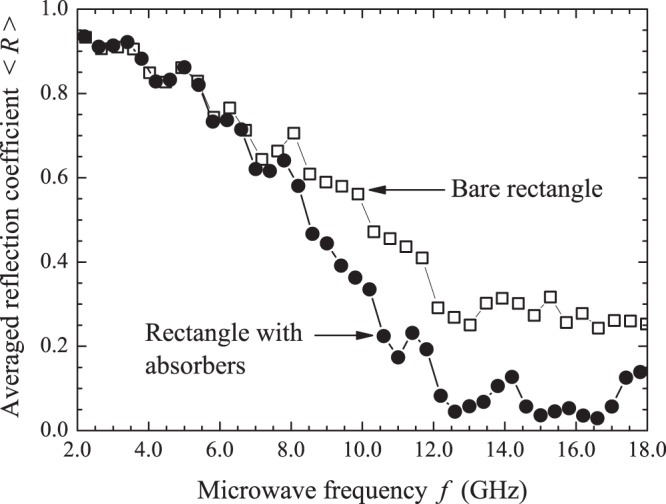
Measured reflection coefficient averaged in a 500 MHz interval centered at frequency *f*, and 80 different antenna positions, for the bare rectangle (open squares) and for the rectangle with strips of microwave absorbers (solid circles). Lines are guides to the eyes.

**Figure 3:**
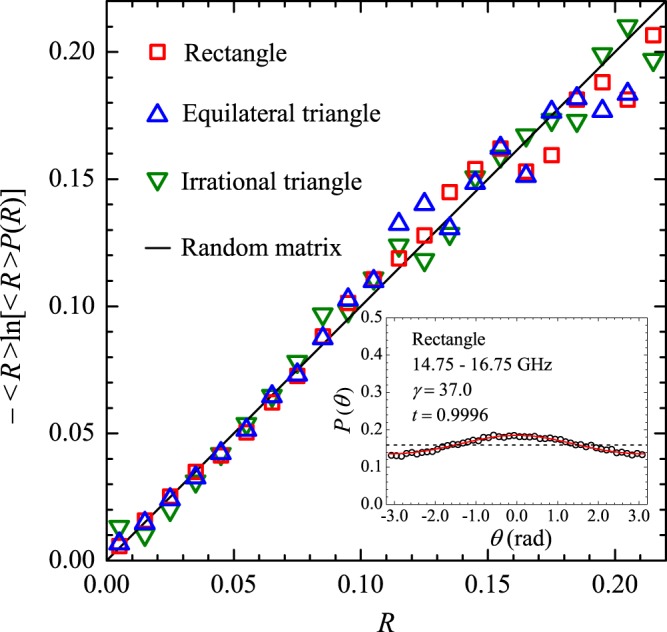
Symbols: Measured distribution *P*(*R*) in the indicated billiard in the strong absorption regime (cavities with absorbers). Frequency intervals are, in GHz, [14.75, 16.75], [16.2, 16.7] and [15.7,16.2], respectively, for the rectangle $(\langle R\rangle =0.0343)$, the irrational triangle $(\langle R\rangle =0.0345)$, and the equilateral triangle $(\langle R\rangle =0.0297)$. Solid line: Exact result predicted by RMT for chaotic scattering. Inset: Symbols: Measured *P*(*θ*) in the rectangle in the same regime. Solid red line: Fit with Eq. (1) with $\gamma =37.0$ and $t=0.9996$. The horizontal dashed line is the Poisson kernel for ideal coupling in the absence of absorption, $P(\theta )=1/2\pi =0.159\ldots$.

**Figure 4:**
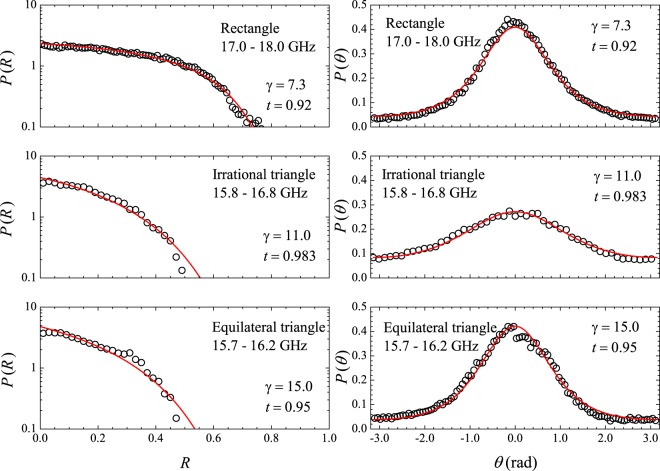
Symbols: Measured distributions *P*(*R*) (left panels) and *P*(*θ*) (right panels) in the indicated billiards and frequency ranges in the Ericson regime. Solid lines are fits with Eq. (1), for the indicated values of parameters *γ* and *t*.

**Figure 5:**
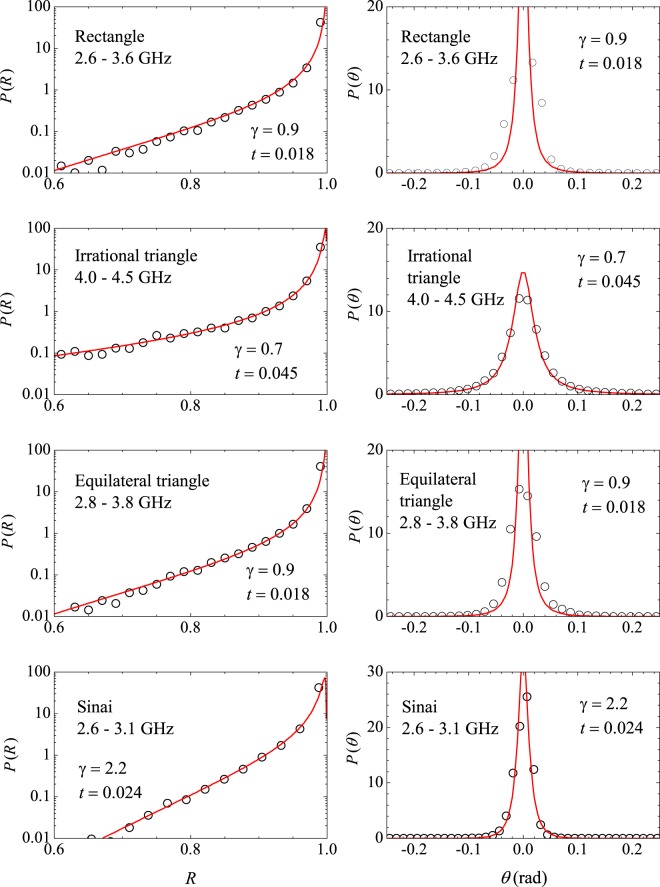
Same as Fig. 4, in the low frequency regime. Parameters *γ* and *t* are for the red solid lines as before. Measurements in a Sinai billiard are shown in the bottom panels.

Typical reflection spectra are shown in Fig. 1. The dots in the upper left spectrum represent the exact eigenfrequencies of the rectangle in the 2–2.5 GHz interval, showing small shifts in the observed resonances for that particular antenna position. As expected, cavity loss precludes the observation of two very close resonances, such as the doublet around 2.1 GHz. As in previous experiments^7^, we first average the measured *R*(*f*) in a small interval (500 MHz) centered at frequency *f* and then in tens of different antenna positions, namely, 80, 40 and 82 for the rectangle, the irrational triangle and the equilateral triangle, respectively. Typical results of the measured $\langle R\rangle \times f$ are shown by the symbols in Fig. 2, open squares for the bare rectangle and solid circles for the rectangle with a set of four strips of absorbers placed on the thin cavity walls. To extract universal characteristics, one must seek intervals where $\langle R\rangle$ is nearly constant. The symbols in Fig. 3 represent the measured probability distribution *P*(*R*) in the strong absorption regime. The frequency intervals considered were, in GHz, $[14.75,16.75]$, $[16.2,16.7]$ and $[15.7,16.2]$, for the rectangle, the irrational triangle and the equilateral triangle, respectively. The same absorber-to-cavity volume ratio was used in the experiments. The solid line in Fig. 3 shows the prediction of RMT^17^, namely, $P(R)=\exp (\,-\,R/\langle R\rangle )/\langle R\rangle$. All data clearly fall in the vicinity of the theoretical result. Small deviations from the uniform distribution due to absorption are also observed in the corresponding *P*(*θ*) in this limit. The symbols in the inset in Fig. 3 show the measured phase distribution in the rectangle, in particular. Notice that the data are shifted by a physically irrelevant nonuniversal phase constant and reinjected in the interval [−*π*, *π*], so that the distribution is symmetric about $\theta =0$ (see the Methods section below). Similar results are observed in the other polygonal billiards. The solid line is a fit to the measured *P*(*θ*) with Eq. (1), with parameters $\gamma =37.0$ and $t=0.9996$. For the rectangle, for instance, the measured value of $\langle R\rangle$ gives $1-\langle R\rangle =0.96$, which is in good agreement with the value of *t* extracted from the fitting procedure. The next step was to characterize the fluctuations in the other regimes of coupling and absorption. For empty cavities and 10 GHz < *f* < 18 GHz, we have the intermediate regime, where superposition of the resonances occurs due to losses. The symbols in Fig. 4 show experimental data for *P*(*R*) (left panels) and *P*(*θ*) (right panels) in the indicated billiard and frequency interval in this intermediate regime. The solid red lines are simultaneous fits with Eq. (1) with parameters *γ* and *t* indicated in the figure. Again, a good agreement between experiment and theory was found. Finally, except for the undetected near degeneracies, we observe well defined peaks for $f < 5\,{\rm{GHz}}$. This regime corresponds to the results shown in Fig. 5, where *P*(*R*) is now concentrated in the vicinity of $R=1$, whereas *P*(*θ*) exhibits a sharp peak around zero. Our results are again consistent with previous experiments in chaotic cavities^7,8^. Table 2 shows a comparison between the *t* values obtained from the simultaneous fitting process in Figs 4 and 5 and those directly measured from $\langle R\rangle$. The ratio $t/(1-\langle R\rangle )$ is close to 1, except in the low-frequency range of the irrational triangle, for which *t* is twice as large. Notice that both *t* and $1-\langle R\rangle$ in this frequency range are very small, and the joint distribution is quite sensitive to the values of *γ* and *t*. Given the hard experimental work underlying the values of *t* and $\langle R\rangle$, we can say that an overall reasonable agreement was found. For completeness, we performed measurements in an asymmetric Sinai billiard, by placing a 8.5-cm-diameter aluminum disc in 45 different positions in the lower right quadrant of the same $41\times 29\,{{\rm{cm}}}^{2}$ rectangular cavity. The results in the 2.6–3.1 GHz interval are shown by the symbols in the bottom panels in Fig. 5, which were successfully simultaneously fitted with Eq. (1) (solid lines), as before.

**Table 2:** Comparison between the value of the coupling parameter *t* extracted from the simultaneous fittings in Figs 4 and 5 and the ones obtained independently from the measured values of $1-\langle R\rangle$.

Billiard	Frequency range (GHz)	Coupling parameter *t*	Measured 1 − 〈*R*〉	Ratio *t*/(1 − 〈*R*〉)
Rectangle	17.0–18.0	0.920	0.74	1.24
Irrational triangle	15.8–16.8	0.983	0.84	1.17
Equilateral triangle	15.7–16.7	0.950	0.84	1.13
Rectangle	2.6–3.6	0.016	0.0164	0.98
Irrational triangle	4.0–4.5	0.045	0.0213	2.11
Equilateral triangle	2.8–3.8	0.020	0.0160	1.25

The number of different antenna positions used in the averaging process was 80, 40 and 82, respectively, for the rectangle, the irrational triangle and the equilateral triangle.

**Figure 6:**
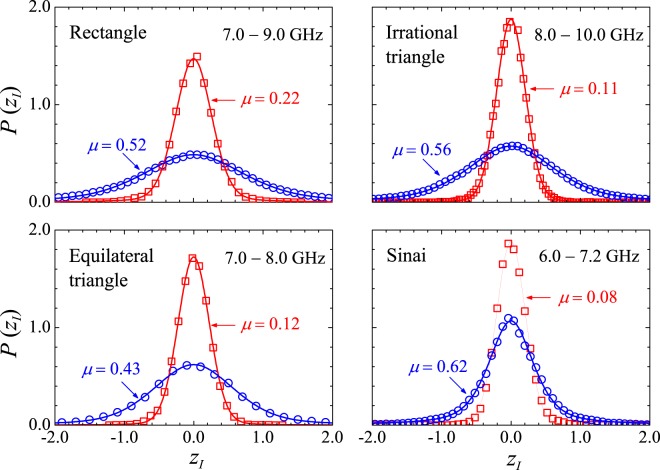
Symbols: Measured *P*(*z*_*I*_) in the indicated billiards and frequency intervals, for two absorption levels. Open blue circles: Empty cavity. Open red squares: Cavity with absorbers. Solid lines are fits with the pseudo-Voigt distribution, as described in the text.

**Figure 7:**
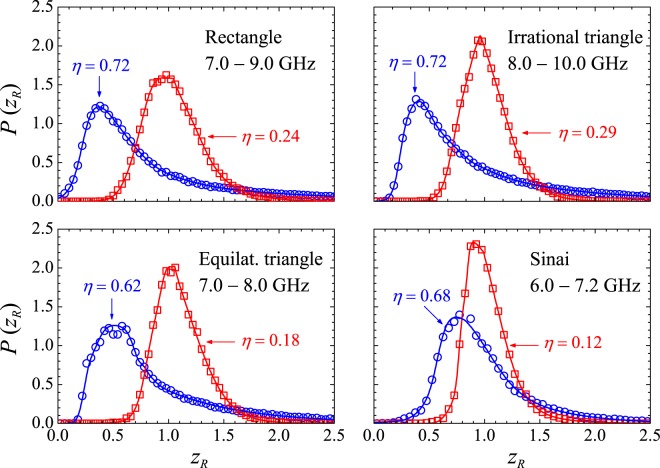
Same as Fig. 6 for *P*(*z*_*R*_). Solid lines are fits with a linear combination of the Chesler-Cram and the asymmetric double-sigmoidal functions, as described in the text.

We further investigated the scattering properties of the polygonal billiards by measuring their normalized impedance. In this case, the scattering statistics no longer depend on coupling, only on the absorption level^10^. The symbols in Figs 6 and 7 show the measured distributions *P*(*z*_*I*_) and *P*(*z*_*R*_), respectively, for two levels of absorption. For comparison, we also show results in the chaotic Sinai billiard. Since a transition from a Lorentzian distribution (low absorption level) to a Gaussian distribution (high absorption level) has been predicted for the imaginary part in quantum chaotic systems^9,10^, the pseudo-Voigt form $P(x)=\mu L(x)+(1-\mu )G(x)$, where *G*(*x*) and *L*(*x*) are the Gaussian and Lorentzian distributions, respectively, was used to fit the observed *P*(*z*_*I*_) (Fig. 6). The weight parameter *μ* for the blue (red) line is 0.52 (0.22), 0.56 (0.11), 0.43 (0.12) and 0.62 (0.08), for the rectangle, irrational triangle, equilateral triangle and chaotic billiard, respectively, clearly demonstrating a transition from a distribution with a stronger Lorentzian fingerprint at lower absorption levels. To fit *P*(*z*_*R*_), whose peak shifts to the right with increasing absorption, as predicted by random matrix theory for chaotic geometries, we used a weighted linear combination $P(x)=\eta C(x)+(1\,-\,\eta )S(x)$ of the Chesler-Cram (CCE) function

5 $$C(x)={A}_{C}\{{e}^{-\tfrac{{(x-{x}_{1})}^{2}}{2w}}+B\{1-\tfrac{1}{2}\{1-\,\tanh \,[{k}_{2}(x-{x}_{2})]\}\}{e}^{-\tfrac{1}{2}{k}_{3}[|x-{x}_{3}|+(x-{x}_{3})]}\},$$

and the asymmetric double-sigmoidal (A2*σ*) function,

6 $$S(x)={A}_{S}\{\tfrac{1}{1+\exp \,[-(x-{x}_{c}+\tfrac{{w}_{1}}{2})/{w}_{2}]}\}\,\{1-\tfrac{1}{1+\exp \,[-(x-{x}_{c}-\tfrac{{w}_{1}}{2})/{w}_{3}]}\},$$

which are commonly found in chromatography, spectroscopy, as well as in biological and geological models. The CCE model combines Gaussian, exponential and hyperbolic tangent functions. The front part of the peak is dominated by the Gaussian (amplitude *A*_*C*_, midpoint *x*_1_, variance *w*), whereas the elongated profile of the tail is described by the combination of the other two functions. On the other hand, the A2*σ* function has full width of half maximum *w*_1_, variance of left side *w*_2_ and variance of the right side *w*_3_. It is symmetric when ${w}_{2}={w}_{3}$, centered at *x*_*c*_. Table 3 displays the parameters used in the curves of Fig. 7. The large number (14) of fitting parameters is certainly detrimental to computational performance, but the idea here is to have a good guide to the eyes through a single function which, indeed, successfully fits all measured normalized resistance distributions.

**Table 3:** Fitting parameters of the curves identified by *η* in Fig. 7.

Billiard	*η*	*A*_*C*_	*x*_1_	*w*	*B*	*k*_2_	*x*_2_	*k*_3_	*x*_3_	*A*_*S*_	*x*_*c*_	*w*_1_	*w*_2_	*w*_3_
Rectangle	0.72	0.35	0.33	0.03	1.11	4.65	0.96	0.90	0.86	3.93	0.49	0.54	0.05	0.16
Rectangle	0.24	0.96	1.19	0.02	2.41	8.54	0.62	6.73	1.04	1.86	0.97	0.39	0.06	0.22
IT	0.72	0.39	0.41	0.01	1.24	4.42	0.93	1.13	0.98	3.81	0.51	0.56	0.03	0.14
IT	0.29	0.62	1.24	0.05	7.80	9.74	0.71	5.11	0.96	1.29	1.05	0.31	0.05	0.08
ET	0.62	0.81	0.28	0.003	1.01	21.9	0.75	1.44	0.87	3.36	0.53	0.40	0.03	0.04
ET	0.18	1.08	0.97	0.002	5.70	10.9	0.80	6.21	1.06	1.89	1.13	0.30	0.09	0.15
Sinai	0.68	0.15	0.48	0.009	17.5	1.68	1.46	2.57	0.82	4.61	0.80	0.45	0.05	0.16
Sinai	0.12	1.98	0.74	0.006	3.79	34.4	0.85	4.05	0.99	1.72	0.97	0.39	0.02	0.10

IT = Irrational Triangle, ET = Equilateral Triangle.

In sum, the measured statistical properties of both *S* and *z* matrices exhibited no distinctive feature when we compare the results in chaotic and in polygonal billiards at room temperature. The observed distributions reported here also show a remarkable similarity with previous results observed in chaotic geometries^6–8,10,11^. This similarity is intriguing and leads to the question: Are the statistical properties of the one-port scattering process independent of the spectral level distribution of the target also in the lossy cavity?

## Discussion

**Figure 8:**
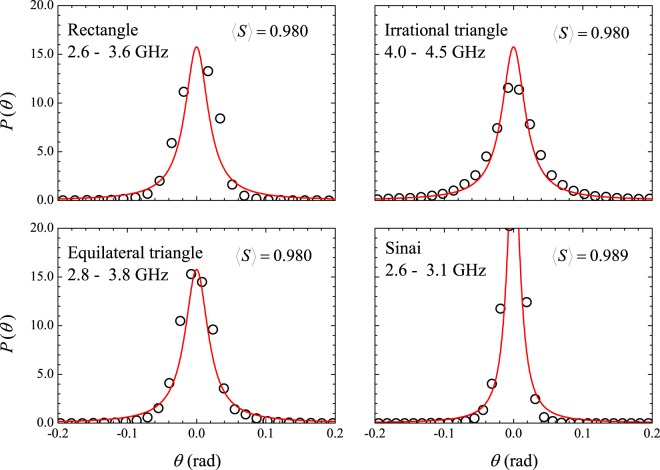
Symbols: Measured phase distributions also shown in Fig. 5. Solid lines are plots of the López-Mello-Seligman formula *P*_LMS_(*θ*) (Eq. (2)), with the indicated values of parameter $\langle S\rangle$.

The question raised above meets a positive answer in the literature when one considers the ideal limit of a cavity with no loss. As mentioned before, according to the analytic-ergodic ensemble of matrices $S={e}^{i\theta }$ introduced by LMS^13^ almost four decades ago, the corresponding phase distribution would be uniquely given by Eq. (2) for both Wigner and Poisson spacing distributions. In our real-world experiments, the cavity losses are small at lower frequencies and it is worth trying to fit our measured phase distribution in that regime with the one-parameter $(\langle S\rangle )$ formula of LMS. The results are shown in Fig. 8, with a reasonable agreement. On the other hand, the fluctuations in the impedance of the lossless cavity have also been predicted to be the same for the Wigner and Poisson spacing distributions in the random wave model of ZAO^12^. The situation at higher absorption levels is intriguing and deserves a deeper analysis. One may ask as to whether the experimental setup could unavoidably introduce chaotic features that compromise the interpretation of the measured distributions. For instance, the antenna might act as a scattering center, effectively changing the boundary of the billiard, particularly at higher frequencies, if the wavelength becomes comparable to the antenna size. From Table 1, we see that in our experiments the smallest wavelength is larger than 16 mm, whereas the antenna has a diameter of only 0.94 mm. In addition, there is no coupling if the antenna is placed on a nodal line of a particular resonant mode. To circumvent these problems, the usual recipe is to average the measured quantities, by varying the antenna position. On the other hand, poor resolution due to losses often prevents the correct level count at small distances. As far as the normalized impedance statistics are concerned, one must bear in mind that absorption alone might be held responsible for a false evidence, if any. A key feature in the experiments, for both the *S* and *z* matrices, is that the frequency interval Δ*f* used for the statistical sampling of the scattering quantities of interest is relatively short, namely, 0.5 GHz ≤ Δ*f* ≤ 2.0 GHz. It is certainly long enough for a measurement of a scattering distribution. However, given the experimental limitations just mentioned, one might ask whether Δ*f* is sufficiently representative for a distinction between correlated and uncorrelated spectra. We will discuss these possible shortcomings based on additional experimental data for three different values of the antenna height, namely, *h* = 1.0, 4.1 and 6.0 mm, and two temperatures $T=77$ and 293 K.

**Figure 9:**
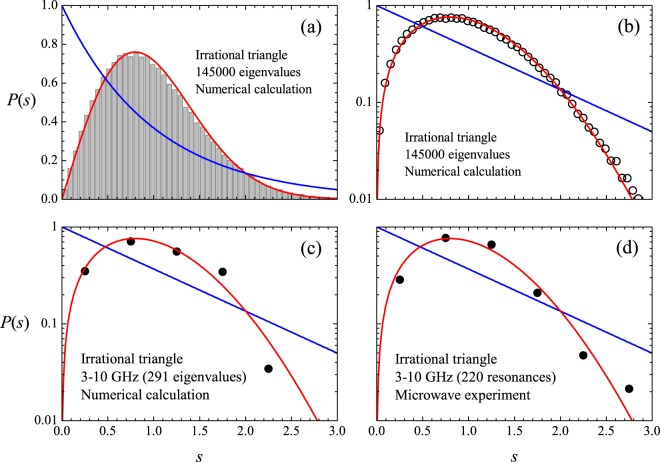
(**a**) Histogram: Numerically calculated nearest neighbor distribution *P*(*s*) in the unfolded spectrum of the irrational triangle, for the first 145000 energy eigenvalues above the first 5000 ones. (**b**) Symbols: Same as (**a**), in a logarithmic scale. (**c**) Symbols: Same as (**a**) for the 291 eigenvalues covered by the 3–10 GHz experimental range. (**d**) Symbols: Measured *P*(*s*) averaged in 6 antenna positions in the 3–10 GHz frequency range, for which only 220 resonances have been observed on average. Blue (red) solid line is the Poisson (Wigner) distribution.

Firstly, let us consider the irrational triangle^15^ for which several spacing distributions are displayed in Fig. 9. The red and blue solid lines are the Wigner (*P*_*W*_(*s*)) and Poisson (*P*_*P*_(*s*)) distributions, respectively. The distributions were obtained from the first *N*_*m*_ eigenvalues above the first *N*_0_ ones. Numerical calculations are shown in the top left panel for ${N}_{0}=5000$ and ${N}_{m}=145000$, where a good agreement with the Wigner distribution is observed. To emphasize this agreement, the same result is shown in the top right panel in a logarithmic scale. Also in a logarithmic scale, the bottom left panel in Fig. 9 shows numerical results obtained in the restricted interval of 3–10 GHz, which corresponds to ${N}_{0}=24$ and ${N}_{m}=291$, chosen for a comparison with experimental results. In this case, we are much closer to the ground state and far from the semiclassical regime. The bottom right panel in Fig. 9 shows the measured *P*(*s*) in the same frequency interval, but averaged in six antenna positions, with $h=4.1\,{\rm{mm}}$ and $T=293\,{\rm{K}}$. Resonances overlooked due to nodal lines and the loss at small spacings due to absorption are responsible for observation of only 220 resonances on average, out of the calculated 291. Despite the restrictions posed by the relatively small values of *N*_0_ and *N*_*m*_, the experimental data are still close to the GOE result. To avoid binning artifacts, it is interesting to consider the cumulative spacing function

7 $$I(s)={\int }_{0}^{s}\,p(s^{\prime} )\,{\rm{d}}s^{\prime} ,$$

which gives the number of spacings below *s*. For the GOE one has

8 $${I}_{{\rm{W}}}(s)=1-\exp \,(\,-\,\pi {s}^{2}/4),$$

and for the Poisson case

9 $${I}_{{\rm{P}}}(s)=1-\exp \,(\,-\,s).$$

**Figure 10:**
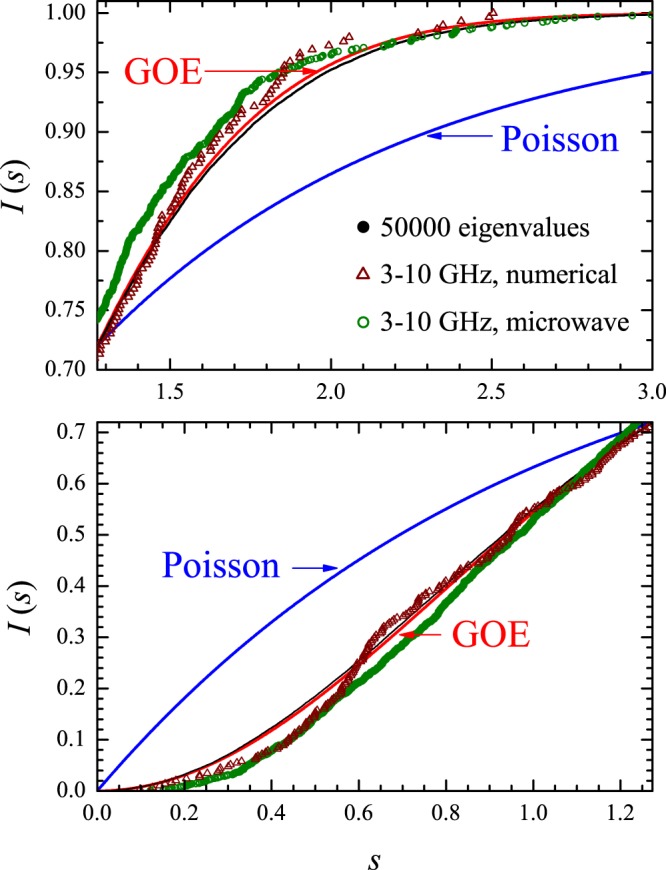
Cumulative spacing functions in the irrational triangular billiard. Solid black circles: Numerical calculations from the first 50000 eigenvalues above the first 50000 ones. Open brown triangles: Numerical calculations in the 3–10 GHz interval. Open green circles: Measured *I*(*s*) in the microwave billiard. Blue (red) solid line is the Poisson (Wigner) theoretical result.

*I*_W_(*s*) and *I*_P_(*s*) are equal for $s=0$, $s=\infty$ and $s=s\ast =\mathrm{1.27324...}$. ${I}_{{\rm{P}}}(s) > {I}_{{\rm{W}}}(s)$ if $0 < s < s\ast$ and ${I}_{{\rm{P}}}(s) < {I}_{{\rm{W}}}(s)$ if $s\ast  < s < \infty$. Calculated and measured cumulative spacing functions in the irrational triangular billiard are shown by the symbols in Fig. 10. The red (blue) solid line is the GOE (Poisson) result. For clarity, the interval $[0,s\ast ]$ (bottom panel) is shown separately from the interval $[s\ast ,3]$ (top panel). Here, ${N}_{0}={N}_{m}=50000$ in the numerical experiments shown by the solid black circles in Fig. 10, which lie very close to the GOE curve. As expected from the results in Fig. 9, also in the vicinity of *I*_W_(*s*) are the results of the numerical (open brown triangles) and microwave (open green circles) experiments restricted to the 3–10 GHz interval. No degeneracy was found in the spectrum of this irrational triangle. The rectangle is more challenging in this regard and is discussed next.

**Figure 11:**
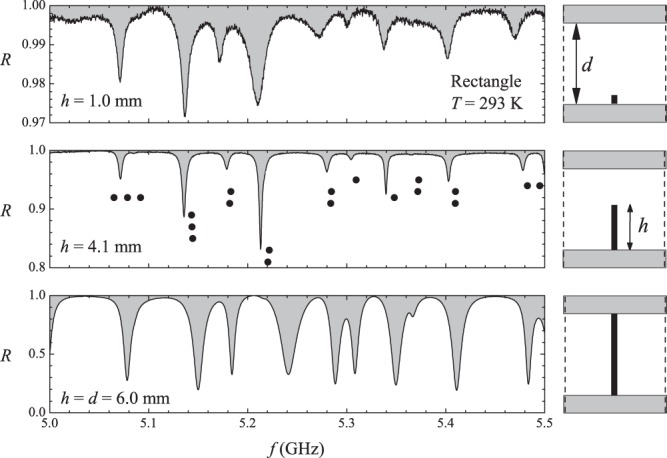
Left panels: Measured reflection coefficient in the rectangle in the 5.0–5.5 GHz frequency range, at room temperature. Right panels: Profile of the top and bottom plates of the cavity with depth *d* and antenna with height *h*. For all cavities, $d=6.0\,{\rm{mm}}$. The values of *h* for the three antennas are indicated in the corresponding left panels. Solid circles in the middle left panel indicate the positions of the exactly calculated resonances.

As is well known^18^, a small metallic bead placed at position $\overrightarrow{\rho }$ inside the cavity disturbs its spectrum through frequency shifts. In other words, a resonance predicted to occur at frequency *f*_0_ actually appears at frequency $f(\overrightarrow{\rho })$ due to the presence of the bead. The frequency shift $\delta f={f}_{0}-f(\overrightarrow{\rho })$ depends on the electromagnetic fields at position $\overrightarrow{\rho }$ in the cavity plane, besides the shape and size of the bead. For a metallic bead in the form of a needle, the frequency shift is approximately proportional to the square of the electric field at position $\overrightarrow{\rho }$. The shift is small if the needle is perpendicular to the electric field and the magnetic contribution is small no matter its direction. The profile of the monopole antenna of height *h* penetrating a cavity of depth *d* is schematically shown in the right panels in Fig. 11. For TM resonating modes, the antenna is parallel to the electric field. In the top panel, the small antenna size (*h* = 1.0 mm) aims at minimizing the perturbation. The corresponding noisy spectrum of the rectangle is shown in the top left panel in the 5.0–5.5 GHz interval. A better coupling is expected for the configuration shown in the middle panel, where *h* is increased to 4.1 mm, which corresponds to a ratio *h*/*d* closer to the commercial value used in coax-waveguide adapters. Indeed, as shown in the middle left panel in Fig. 11, there is a substantial increase in the signal-to-noise ratio. However, notice that these two spectra seem to be statistically equivalent in regard to the spacing distribution. In the bottom right panel, the antenna short-circuits the central conductor of the coaxial cable with the ground at the top cavity plate. With $h=d$, this is the only case that might truly mimic a billiard with a point-like scatterer. Large variations in the absorption level and in the positions of the resonances are observed in this case (bottom left spectrum in Fig. 11). The black solid circles in the middle panel represent the exactly calculated eigenfrequencies, only half of which are observed in the physical experiments in this frequency interval, for that particular antenna position $((x,y)=(10\,{\rm{cm}},7\,{\rm{cm}})$, origin at a corner).

**Figure 12:**
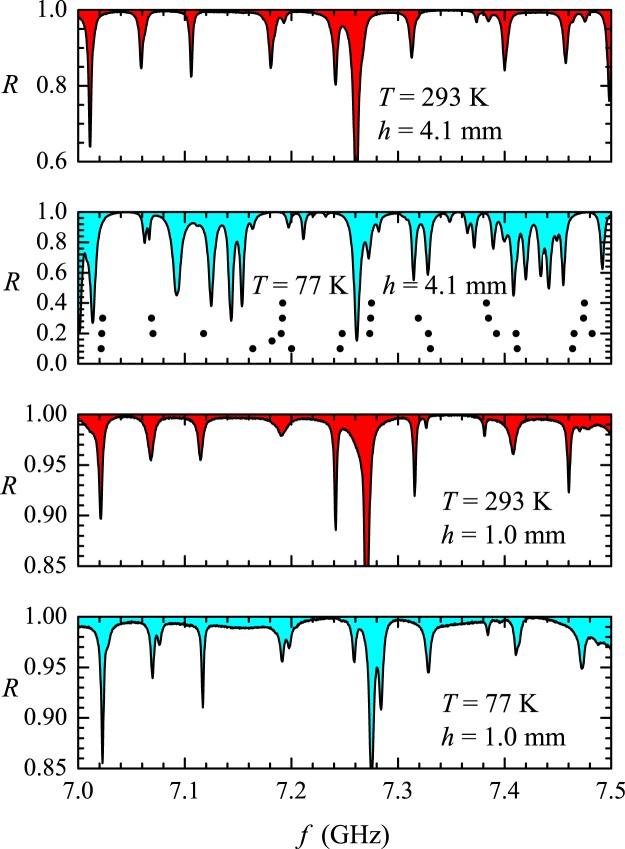
Measured reflection coefficient in the rectangle in the 7.0–7.5 GHz frequency range, for the antennas with $h=1.0$ and 4.1 mm at temperatures $T=293$ and 77 K, as indicated in the corresponding panel. Solid circles in the second panel from top to bottom indicate the positions of the exactly calculated resonances.

On intuitive grounds, one expects that absorption would have a paramount importance in regard to the detection of resonances at small distances. To gain a better understanding of the phenomenon, spectra were also measured with the resonator immersed in a styrofoam box filled with liquid nitrogen. The cavity, here with top and bottom plates with the same size and attached with screws, was mechanically isolated from the bath through a thin partition, but was not evacuated. In our low-cost “cryostat”, thermal stabilization was reached within a couple of hours. The surface resistance in copper falls by a factor around 3 to 4 from room temperature to 77 K at microwave frequencies. The corresponding gain in the quality factor of the resonances gives us some hope of shedding light on the small spacing issue. “Hot” and “cold” reflection spectra in the rectangular billiard are shown in Fig. 12 in the 7.0–7.5 GHz interval for the two smaller antennas. The antennas were placed in exactly the same position $((x,y)=(10\,{\rm{cm}},7\,{\rm{cm}})$, origin at a corner), one at a time. For the antenna with $h=1.0\,{\rm{mm}}$, the splitting of some close resonances are clearly seen in the cold spectrum (bottom panel). More dramatically, the cold spectrum observed with the 4.1-mm high antenna (second panel from top to bottom in Fig. 12) exhibits the splitting of nearly all resonances which are hidden in the hot spectra, as indicated by the solid black circles. Let us see how these changes affect the spacing distribution in the rectangle.

**Figure 13:**
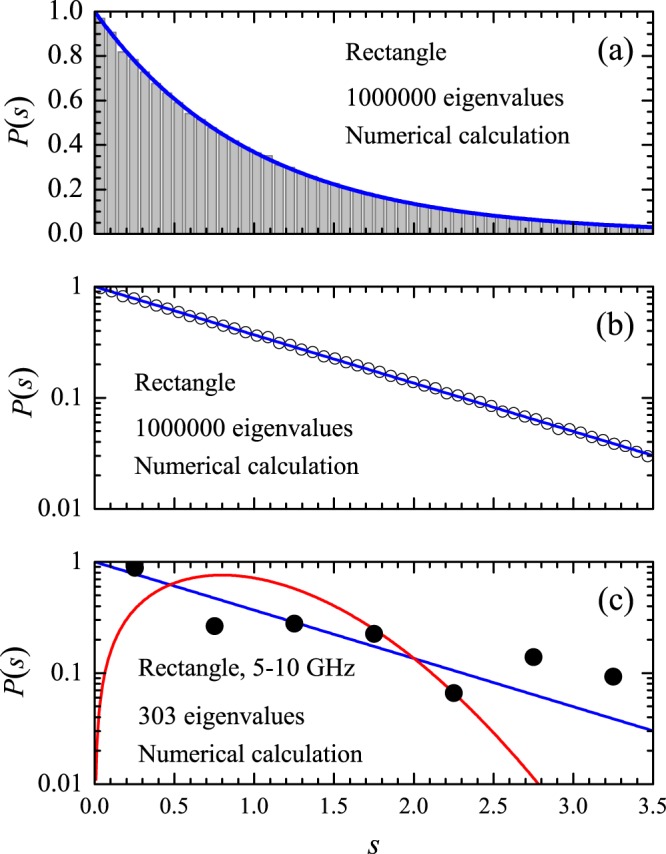
Calculated nearest neighbor spacing distribution *P*(*s*) in the rectangle. (**a**) Histogram: Numerical results from the the first ${N}_{m}=1000000$ eigenvalues beyond the first ${N}_{0}=5000$ ones. Solid line: Poisson distribution *P*_P_(*s*). (**b**) Same as (**a**) in a logarithmic scale. (**c**) Symbols: Numerical results from the first ${N}_{m}=303$ eigenvalues beyond the first ${N}_{0}=93$ ones, corresponding to the experimental interval of 5–10 GHz. Solid blue (red) line is the Poisson (Wigner) distribution.

**Figure 14:**
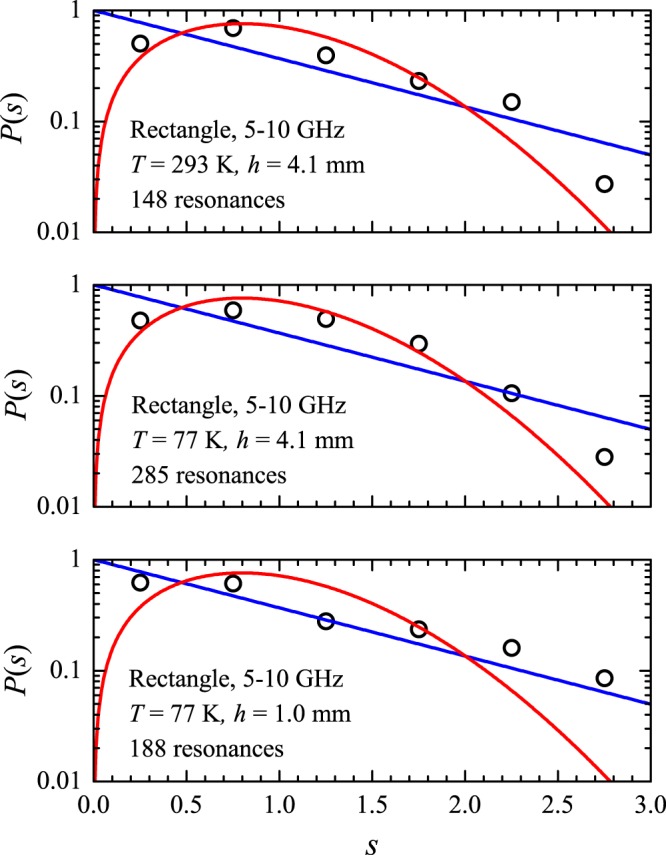
Symbols: Measured nearest neighbor spacing distribution in the rectangle in the 5–10 GHz frequency interval, for the indicated values of antenna height *h* and temperature *T*.

**Figure 15:**
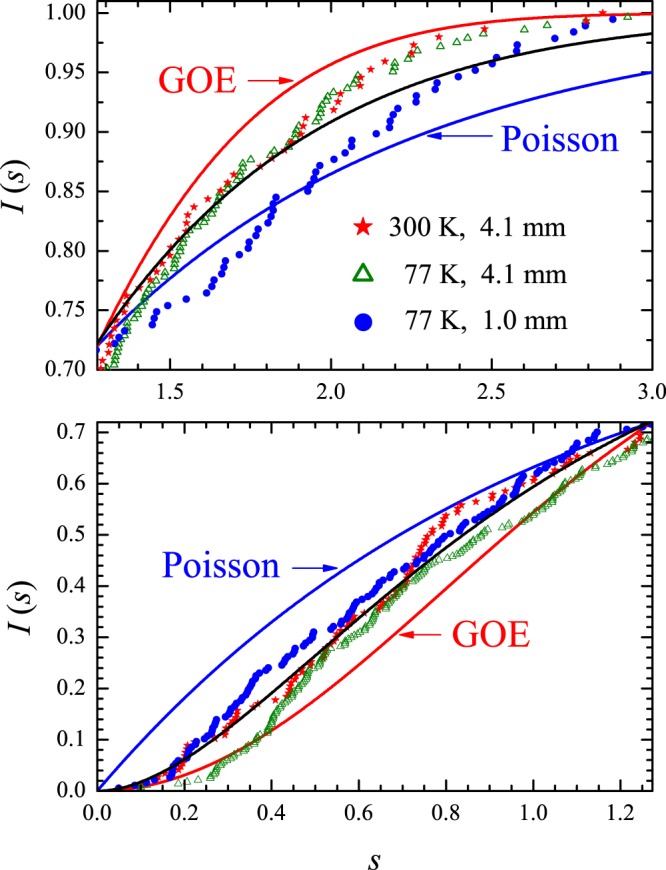
Symbols: Measured cumulative spacing functions in the rectangular billiard with the indicated values of temperature and antenna height. Blue (red) solid line is the Poisson (Wigner) theoretical result. The solid black line is a plot of the semi-Poisson function ${I}_{{\rm{SM}}}(s)=1-[(1+2s)\,\exp (\,-\,2s)]$, for comparison.

The histogram shown in the top panel in Fig. 13 is the spacing distribution in the spectrum of the rectangular cavity calculated with ${N}_{0}=5000$ and ${N}_{m}=1000000$, showing a remarkable agreement with the Poisson distribution, as expected. The symbols in the middle panel show the same calculated distribution in a logarithmic scale. The symbols in the bottom panel were calculated in the restricted 5–10 GHz interval, which corresponds to ${N}_{0}=93$ and ${N}_{m}=303$. In spite of the limitations due to the small values of *N*_0_ and *N*_*m*_, a signature of the semiclassical distribution in the calculated *P*(*s*) can still be observed. For comparison, three measured spacing distributions for the same antenna position as above are shown by the symbols in Fig. 14. At room temperature and for $h=4.1\,{\rm{mm}}$ (top panel), only 148 resonances are observed in the experiments out of the exactly calculated 303. As a result, the Poissonian fingerprint is missing in the measured histogram. For $T=77\,{\rm{K}}$ and $h=4.1\,{\rm{mm}}$ (middle panel), the number of resonances increases to 285. However, due mostly to large frequency shifts in the splitting of the multiplets, the measured distribution is not as close to the blue line as it is in the bottom panel in Fig. 13. Finally, the symbols in the bottom panel in Fig. 14 show the measured distribution at $T=77\,{\rm{K}}$ with $h=1.0\,{\rm{mm}}$. The trend towards the Poisson statistic is more evident now, even with a reduced number (188) of observed resonances, and is confirmed by the measured cumulative spacing functions shown by the symbols in Fig. 15. Notice that by reducing even further both the antenna size $(h\approx 0.5\,{\rm{mm}})$ and the losses, experiments in superconducting microwave billiards have indeed exhibited the Poisson distribution in integrable geometries^19–21^. At room temperature, the role of absorption has been pointed out in the pioneering experiments by Stöckmann and Stein^22^. The apparent level repulsion observed was later associated with a pseudointegrability of a billiard with a point-like scatterer^23^. More recently^24^, a Green’s function approach has shown that the rectangle with a point-like scatterer indeed exhibits a Poissonian *P*(*s*) in the semiclassical regime, thus conciliating the quantum and classical results. Intermediate long-range correlations have also been observed experimentally in rectangular microwave billiards^25^.

**Figure 16:**
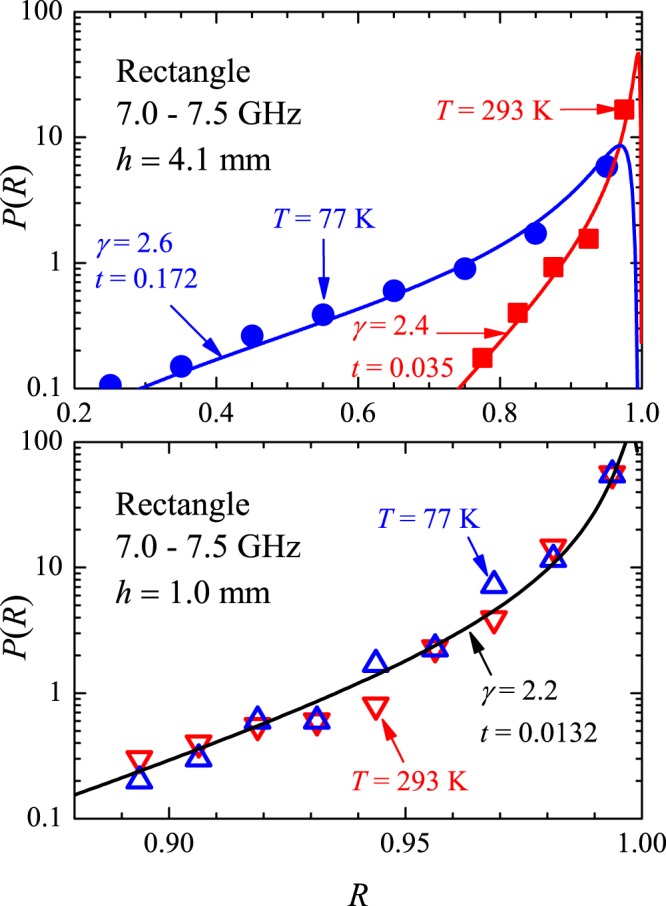
Top panel: Measured distribution of reflection coefficient in the 7–7.5 GHz interval for the antenna with $h=4.1\,{\rm{mm}}$ at temperature $T=77\,{\rm{K}}$ (blue circles) and $T=293\,{\rm{K}}$ (red squares). Lines are fits with Eq. (1) for the indicated values of parameters *γ* (absorption) and *t* (coupling). Bottom panel: Same as the top panel, for the antenna with $h=1.0\,{\rm{mm}}$.

Finally, distributions of the reflection coefficient for a single antenna position (no averaging) and two temperatures are displayed in Fig. 16, for $h=4.1\,{\rm{mm}}$ (top panel) and for *h* = 1.0 mm (bottom panel), in the 7.0–7.5 GHz interval, in the rectangle. All data could again be fitted with Eq. (1). Notice that there is no significant difference between the *hot* and *cold* distributions observed with $h=1.0\,{\rm{mm}}$, despite the splittings which are present in the spectrum at 77 K, but absent at room temperature.

**Figure 17:**
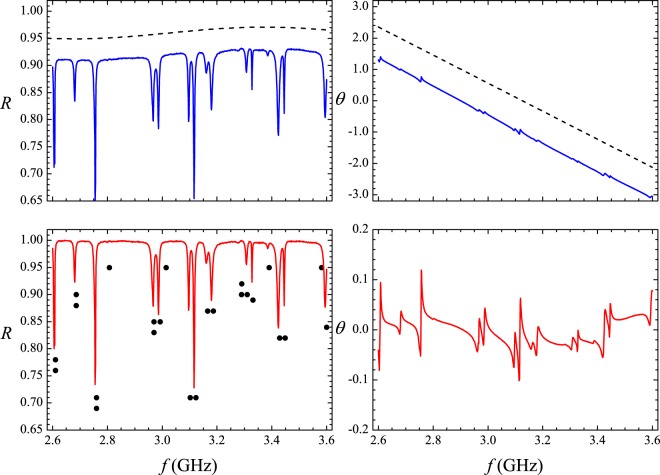
Subtraction of backgrounds at lower microwave frequencies for the measurement of the reflection coefficient *R* (left panels) and the phase *θ* of the *S* matrix (right panels), for a single antenna position in the rectangle. Top panels: Solid blue lines: Measured signals. Dashed black lines: upwardly shifted backgrounds, sinusoidal for *R*, linear for *θ*. Bottom panels: Solid red lines: Measured signals subtracted by the corresponding background. The black dots in the lower left panel indicate the calculated resonances. In this case, due to the proximity of the antenna to nodal lines and limited resolution, only 16 of the 24 predicted resonances are experimentally detected. Six nearby resonances are missing due to absorption, two due to poor coupling (antenna close to a nodal line). Otherwise, calculated and observed eigenfrequencies are in good quantitative agreement.

### Conclusive remarks

In sum, we have presented results from a thorough experimental study of the one-port microwave scattering in billiards with four different geometries, both classically chaotic and nonchaotic, at room temperature. All measured *scattering* statistics (reflection coefficient, phase of the *S*-matrix, normalized resistance, and normalized reactance) are found to be independent of geometry, in all levels of coupling and absorption. For low absorption levels, the results are consistent with the theoretical predictions by López, Mello and Seligman, for the phase distribution of the analytic-ergodic ensemble of random *S* matrices, and by Zheng, Antonsen and Ott, for the normalized impedance of the lossless cavity within the random wave model. Both theories predict that the corresponding scattering distribution is the same for both Wigner and Poisson distributions of level distances. In other words, there seems to be no clear-cut signature of chaos in the scattering statistics in that limit. On the other hand, the nonuniversal effects of a particular coupling are removed in the normalized impedance approach. Here, we also observe a remarkable similarity between the scattering properties of chaotic and nonchaotic geometries. Additional experiments for three different sizes of the antenna and two different temperatures, 77 K and 293 K, confirm that the effects due to losses are severe in the characterization of the *spectral* properties, but do not indicate qualitative changes in the *scattering* properties. An explanation for the observed uniqueness of the measured scattering statistics at room temperature may reside in the similarity of the spectra due to low resolution, combined with the relatively short frequency intervals (0.5 GHz ≤ Δ*f* ≤ 2.0 GHz) on which the statistical sampling of *R*, *θ*, *z*_*R*_ and *z*_*I*_ are based. In all cases, the measured distributions *P*(*R*), *P*(*θ*), *P*(*z*_*R*_) and *P*(*z*_*I*_) are in agreement with previous predictions from random matrix theory for the lossy cavity. Theoretical and numerical studies that could shed light on the differences between the scattering properties of chaotic and nonchaotic systems are still missing. In the meantime, further experiments at cryogenic temperatures are under way.

## Methods

### Setup

Our home-made resonators are comprised of two polished copper plates that bracket 6.0-mm thick bars which define the planar geometry. For the excitation of the cavity, we use a standard coaxial-line probe antenna, consisting of a commercial SMA female two-hole flange mount connector with gold plated solder cup terminal, with an external diameter of 1.22 mm (Fairview Microwave, model SC7486). A small piece of 0.94-mm diameter wire is welded at the cup terminal so that the inner conductor of the coaxial connector is extended a total height of 4.1 mm into the cavity, perpendicularly to the much larger bottom plate ($1200\times 600\times 2\,{{\rm{mm}}}^{3}$), which rests on the *xy* plane of a thick wooden table. A hole was drilled in the table, so that the antenna, fixed at the center of the bottom plate, could be reached by a flexible coaxial cable from below. The walls of the cavity and the upper Cu plate are fixed by a weight (60 kg) uniformly distributed on the top of the structure, thus defining the position of the antenna relative to the side walls. The incoming radiation is coupled with the electric field of TM resonant modes of the cavity, which are two-dimensional for frequencies below 25 GHz. The complex matrices *S* and *z* were measured in tens of different antenna positions in each billiard, with a vector network analyzer (Anritsu, model 37247D VNA, 40 MHz–20 GHz) in the 2–18 GHz frequency range, divided into 8 intervals of 2 GHz. Each measurement (2–4 GHz, 4–6 GHz, 6–8 GHz, 8–10 GHz, 10–12 GHz, 12–14 GHz, 14–16 GHz and 16–18 GHz) was recorded with a discretization interval of 1.25 MHz, and properly calibrated with precision devices (Anritsu N Calibration Kit, model 3653, which includes Short (Anritsu, model 23N50), Open (Anritsu, model 24N50) and Termination (50 Ω, Anritsu, model 28N50-2) loads). The monopole antenna and the VNA are connected through a flexible type-N coaxial cable (Anritsu, model 3670N-2) and a N female - SMA male coaxial adapter (Fairview Microwave, model SM4265). The microwave absorbers used in the experiments are light-weight flexible polyurethane foam material (Emerson & Cuming Microwave Products, ECCOSORB LS-26/SS-3 (0.8–18 GHz)), held in place by a pressure sensitive adhesive. The data were recorded in a desktop computer through a National Instruments GPIB-USB-HS interface.

### Baseline corrections

The coupling of the cavity with the power supply through adapters and cables leads to a frequency dependent background in the reflected signals. This background must be subtracted before an analysis is made. In the low-frequency range, a set of points not influenced by the resonances were selected and fitted with a simple function *bg*(*f*). Each point of the measured signal was subsequently subtracted by *bg*(*f*). For instance, the blue solid lines in the top panels in Fig. 17 show the original signals *R*(*f*) (left) and *θ*(*f*) (right) for 2.6 GHz < *f* < 3.6 GHz in the rectangle. Shifted upwards for clarity, the dashed black lines show a sinusoidal *bg*(*f*) in the reflection coefficient signal (top left panel) and a linear *bg*(*f*) in the phase signal (top right panel). The solid red lines in the bottom panels in Fig. 17 are the corresponding signals after subtraction of the corresponding *bg*(*f*).

### Fittings

As far as the fittings with Eq. (1) are concerned, we follow Kuhl *et al*.^26^. The interpolation formula is given by

10 $$P(R,\theta ;\gamma ,t=1)={C}_{1}\tfrac{{e}^{-\alpha /(1-{R}_{0}(R,\theta ))}}{{(1-{R}_{0}(R,\theta ))}^{5/2}}[A{\alpha }^{-1/2}+B{(1-{R}_{0}(R,\theta ))}^{1/2}],$$

where $$\alpha =\gamma /2$$, $$A=\alpha ({e}^{\alpha }\,-\,1)$$, $$B=1+\alpha -{e}^{\alpha }$$, $${C}_{1}=1/[A{\rm{\Gamma }}(3/2,\alpha )/{\alpha }^{2}+B{e}^{-\alpha }/\alpha ]$$ and $${\rm{\Gamma }}(x,\alpha )=$$$${\int }_{\alpha }^{\infty }\,{t}^{x-1}{e}^{-t}dt$$ is the upper incomplete gamma function. In addition, $${R}_{0}=|{S}_{0}{|}^{2}$$, $${S}_{0}=(S-\langle S\rangle )/(1-\langle S\rangle S)$$, $$\langle S\rangle =\sqrt{1\,-\,t}$$. We use the commercially available software Mathematica to integrate Eq. (1) in a trial and error procedure. Input values for parameters (*γ*, *t*) could be found in the literature^7^. A guess for *t* might also be obtained from the measured value of $$1-\langle R\rangle $$. Firstly, we adjust *γ* and *t* to fit *P*(*R*) and from them new values were found to fit *P*(*θ*), which were then used as new input to fit *P*(*R*) and so forth, until good agreement to both experimental distributions was found. The goodness of the fittings was quantified by numerical tests also provided by Mathematica, through the command DistributionFitTest[“data”, “dist”, ‘property”].

## Data Availability

The datasets generated during the current study are available from the corresponding author on reasonable request.
